# Accumulation of organic C components in soil and aggregates

**DOI:** 10.1038/srep13804

**Published:** 2015-09-11

**Authors:** Hongyan Yu, Weixin Ding, Zengming Chen, Huanjun Zhang, Jiafa Luo, Nanthi Bolan

**Affiliations:** 1 State Key Laboratory of Soil and Sustainable Agriculture, Institute of Soil Science, Chinese Academy of Sciences, Nanjing 210008, China; 2 School of Environment and Civil Engineering, Jiangnan University, Wuxi 214122, China; 3 Land and Environment, AgResearch, Hamilton 3240, New Zealand; 4 Centre for Environmental Risk Assessment and Remediation, University of South Australia, SA 5095, Australia; 5 Global Centre for Environmental Remediation, University of Newcastle, NSW 2308, Australia

## Abstract

To explore soil organic carbon (SOC) accumulation mechanisms, the dynamics of C functional groups and macroaggregation were studied synchronously through aggregate fractionation and ^13^C NMR spectroscopy in sandy loam soil following an 18-year application of compost and fertilizer in China. Compared with no fertilizer control, both compost and fertilizer improved SOC content, while the application of compost increased macroaggregation. Fertilizer application mainly increased the levels of recalcitrant organic C components characterized by methoxyl/N-alkyl C and alkyl C, whereas compost application mainly promoted the accumulation of methoxyl/N-alkyl C, phenolic C, carboxyl C, O-alkyl C and di-O-alkyl C in bulk soil. The preferential accumulation of organic C functional groups in aggregates depended on aggregate size rather than nutrient amendments. These groups were characterized by phenolic C and di-O-alkyl C in the silt + clay fraction, carboxyl C in microaggregates and phenolic C, carboxyl C and methoxyl/N-alkyl C in macroaggregates. Thus, the differences in accumulated organic C components in compost- and fertilizer-amended soils were primarily attributable to macroaggregation. The accumulation of methoxyl/N-alkyl C in microaggregates effectively promoted macroaggregation. Our results suggest that organic amendment rich in methoxyl/N-alkyl C effectively improved SOC content and accelerated macroaggregation in the test soil.

The application of organic amendments such as manure, compost and biosolids is a widely adopted strategy to improve the soil organic matter (SOM) levels and increase the sequestration potential of atmospheric carbon dioxide in soils^1^,^2^,^3^. Understanding the variation and predicting the dynamics of carbon stocks in soil following organic amendment application require a thorough understanding of the mechanisms by which organic molecules are stabilized in soil^4^. Soil physical particle size fractionation, combined with chemical and spectroscopic analyses, is a widely used approach to investigate organic molecule dynamics, linking mineral particles and soil structure^5^.

Using the density fractionation method, SOM accumulation was found mainly depending on the preferential accumulation of O-alkyl C or carboxyl/carbonyl groups in the free- and/or occluded light fractions in paddy or grassland soils^6^,^7^, and alkyl C, aliphatic components or microbial-derived organic components (e.g., amide N) in mineral-associated organic matter fractions in forest or upland soils^8^,^9^,^10^. Through particle size fractionation, phenolic C and polysaccharides (O-alkyl C) were found stabilization by the clay fractions, and lignin and its phenol products (methoxyl C and/or phenolic C) were protected by silt minerals^4^,^11^,^12^,^13^.

Through the aggregate fractionation, it was confirmed that the content of O-alkyl C usually decreased with the decrease of aggregate size, however, the ratio of O-alkyl C to aryl C increased^6^,^14^. Intra-aggregate C contained higher concentrations of aromatic, alkyl, and carbonyl groups but a lower abundance of O-alkyl groups than those inter-aggregate C^15^. Following compost or farmyard manure application, the content of humic components and total sugar in macroaggregates (>250 μm) and/or the silt + clay fractions was found to increase in arable soils^16^,^17^,^18^. Biochar application could enrich aromatic-C, carboxyl C and traces of ketones and esters mainly in unprotected organic matter and within aggregates, whereas green manure application enriched conjugated carbonyl-C such as ketones and quinones as well as CH deformations of aliphatic-C mainly in the intra-aggregate fraction in organic C poor soils^19^.

Compared with organic molecules accumulation in particle size and density fractions (primary organo–mineral complexes in bulk soil), their accumulation in aggregates (secondary organo-mineral complexes, the soil structure unit containing primary organo-mineral particles and particles of uncomplexed OM) was less investigated because primary organo–mineral complexes were believed to be responsible for the long-term SOM sequestration^20^. Most investigations on organic molecular characteristics in aggregates still focused on the change of SOM quality for their sensitiveness by management practices. However, the most widely accepted mechanism for SOM accumulation in response to organic amendment application over the course of several decades is that organic amendment application improves soil macroaggregate structure (macroaggregation) to physically protect SOM from biodegradation by microorganisms^21^,^22^,^23^,^24^ Macroaggregation is primarily meditated by plant roots, microbial hyphae and organic or inorganic binding agents in the soil. After organic input, macroaggregation was found to be associated with the biochemical characteristics of the organic products such as water-extractable polysaccharide, cellulose and hemicellulose, and lignin contents^25^. Therefore, elucidating how soil organic carbon (SOC) accumulation occurs after organic amendment application requires the mechanisms of organic component accumulation in aggregates and the formation of macroaggregates to be simultaneously explored on a molecular scale.

In the North China plain, a long-term field experiment was established to monitor the dynamic variation in SOC. Our previous study showed that the SOC increase under compost application mainly occurred in macroaggregate, and macroaggrege (>250 μm) formation was closely correlated with the content of organic carbon in microaggregate (250–53 μm) and the silt + clay fractions (<53 μm)^24^. Enzyme activities analyses implied that the accumulated C components might be lignocellulose and sucrose in macroaggregate, lignocellulose and hemicellulose in microaggregate and lignin (its derivative) and nonstructural carbohydrates in the silt + clay fraction after long-term compost application^18^. Thus, we assumed that compost application possibly accelerated the accumulation of polysaccharide and phenolic C in microaggregate and the silt + clay fractions to promote macroaggregation and then protect O-alkyl C accumulation in macroaggregate. The objectives of the current study were (1) to understand the biochemical characteristics of accumulated organic matter on the aggregate scale and (2) to explore macroaggregate formation on the molecular scale after long-term compost application.

## Results

### Aggregate mass distribution and OC concentration

**Figure 1:**
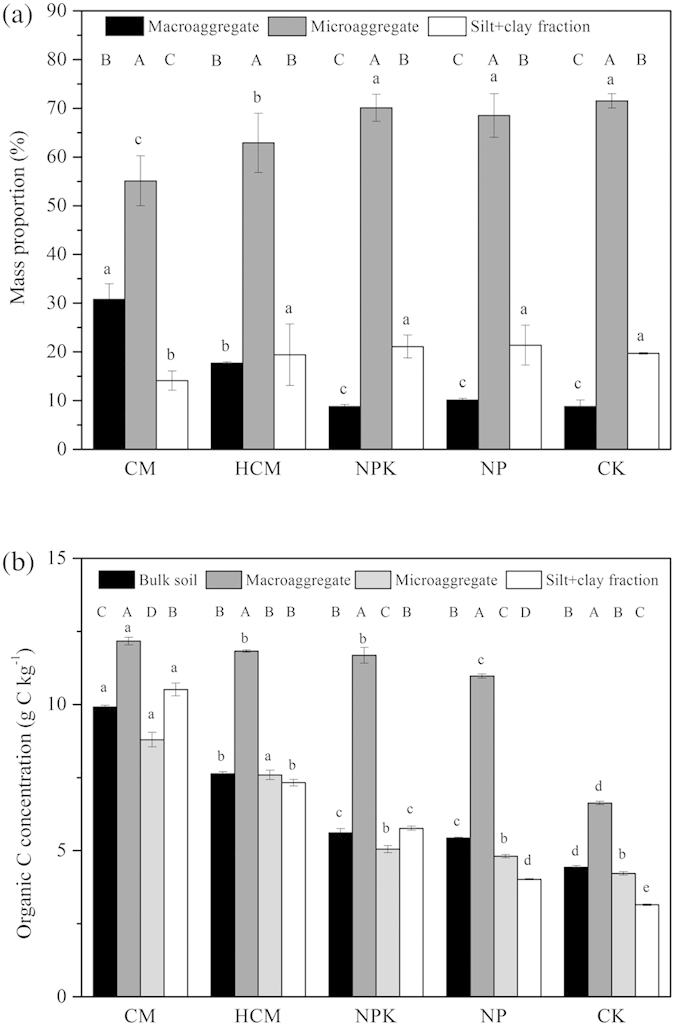
Mass proportion of aggregates (a) and organic C content (b) in bulk soils and aggregates, as affected by long-term application of compost and mineral fertilizers. Different lowercase letters indicate significant differences between treatments for the same aggregate, and different capital letters indicate significant differences between aggregates for the same treatment (Tukey’s test, *P* < 0.05, *n* = 4).

Compost application alone (CM) or in combination with NPK (HCM) significantly increased the mass proportion of macroaggregates (*P* < 0.05, by 250% and 101%, respectively) and reduced the proportion of microaggregates and the silt + clay fractions compared with CK (Fig. 1a). Mineral fertilizers did not significantly affect macroaggregation. Long-term application of compost more effectively improved OC content in bulk soil than did long-term mineral fertilizer application; the percentage increase in OC compared with CK was as follows: CM (124%) > HCM (72%) > NPK (27%) > NP (23%; Fig. 1b). Compared with CK, compost application alone (CM) or in combination with NPK (HCM) increased the OC content in macroaggregates, microaggregates and the silt + clay fractions. Mineral fertilizer amendment also increased the OC content in macroaggregates and the silt + clay fractions but not in microaggregates.

### Accumulation of C functional groups in aggregates

The ^13^C-CPMAS-NMR spectra of bulk soils and aggregates are shown in Fig. S1. The spectra were slightly different among aggregates within the same treatment group or among treatment groups for the same aggregate, which was clearly revealed by the variation in the relative content and content of C functional groups (Tables S1 and S2).

**Figure 2:**
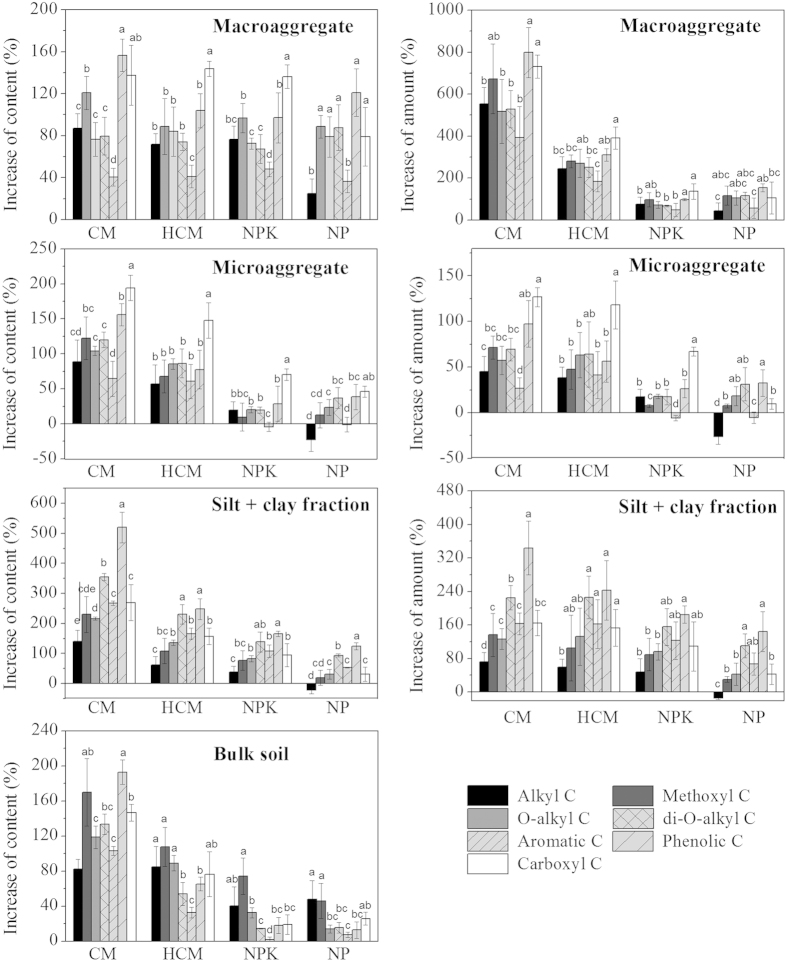
Increase in organic C functional group content or amount in bulk soils and aggregates, as affected by long-term application of compost and fertilizers. Different lowercase letters indicate significant differences between C functional groups for the same treatment and aggregate (Tukey’s test, *P* < 0.05, *n* = 4).

According to the content of C functional groups (Table S2), the increase in phenolic C, carboxyl C and methoxyl/N-alkyl C content (88.8–156.6%) was higher than that of O-alkyl C, di-O-alkyl C, alkyl C (67.3–87.0%) and aromatic C (40.5–48.0%) in macroaggregates in compost- or NPK-amended soils (Fig. 2). Moreover, irrespective of the nature of the amendments, all amendments preferentially promoted the accumulation of carboxyl C in microaggregates (46.0–194.1% vs. −22.9–156.1% for other C functional groups), as well as the accumulation of phenolic C and di-O-alkyl C in the silt + clay fractions (93.5–520.0% vs. −21.4–314.7% for other C functional groups; Fig. 2).

### Effect of compost and mineral fertilizer application on the accumulation of C functional groups

Compost application (CM and HCM) mainly promoted the accumulation of methoxyl/N-alkyl C (increase in content = 107.9–170.2%), phenolic C (65.4–193.1%), carboxyl C (76.5–146.7%), O-alkyl C (89.2–118.8%) and di-O-alkyl C (54.3–133.5%) in bulk soil compared to the CK (Fig. 2). The application of mineral fertilizers mainly increased the contents of methoxyl/N-alkyl C (46.2–74.4%) and alkyl C (40.3–48.2%) in bulk soil.

**Figure 3:**
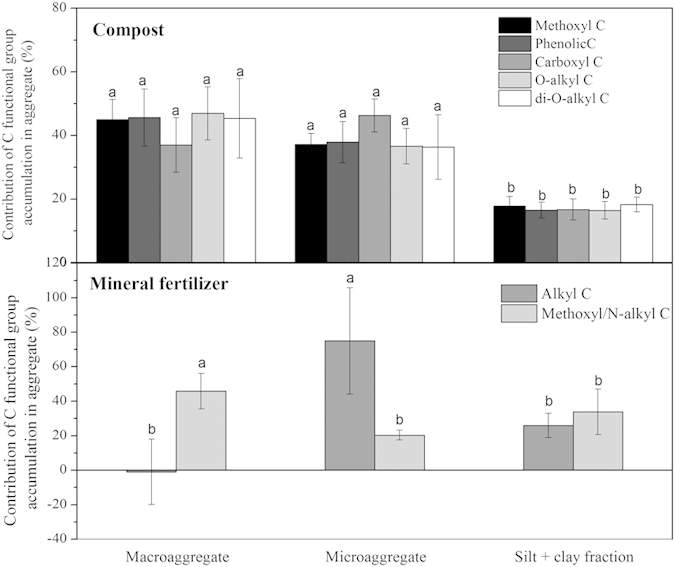
Contribution of the increased amounts of main organic C functional groups in aggregates, as affected by the application of compost (average for HCM and CM) and mineral fertilizer (average for NPK and NP). Different lowercase letters indicate significant differences between aggregates for the same C functional group (Tukey’s test, *P* < 0.05, *n* = 4).

When simultaneously considering the variation in aggregate mass and the increase in C functional group content in compost-amended soils, the increase in the amount of methoxyl/N-alkyl C, phenolic C, carboxyl C, O-alkyl C and di-O-alkyl C occurred in the following order: macroaggregates > the silt + clay fractions > microaggregates (Fig. 2). The average proportion of increased methoxyl/N-alkyl C, phenolic C, O-alkyl C and di-O-alkyl C content in macroaggregates to their corresponding increased values in compost-amended soils (CM and HCM) was 45.0%, 45.6%, 46.9% and 45.4%, respectively, which was higher than those in the silt + clay fractions (significantly) and microaggregates (not significantly). The average proportion of increased levels of carboxyl C in microaggregates in the HCM and CM treatment groups was 46.3%, which was higher than that in macroaggregates (37.0%, not significantly; Fig. 3). In mineral fertilizer-amended soils, the increasing pattern observed for C functional groups was the same as that for their content since the mass proportion of aggregates did not significantly change. The additional alkyl C was mainly distributed in microaggregates, accounting for 78% of the total increase in soil, whereas higher accumulation of methoxyl/N-alkyl C was observed in macroaggregates and the silt + clay fraction (Fig. 3).

### Specific enzyme activities and particle sizes analysis

**Figure 4:**
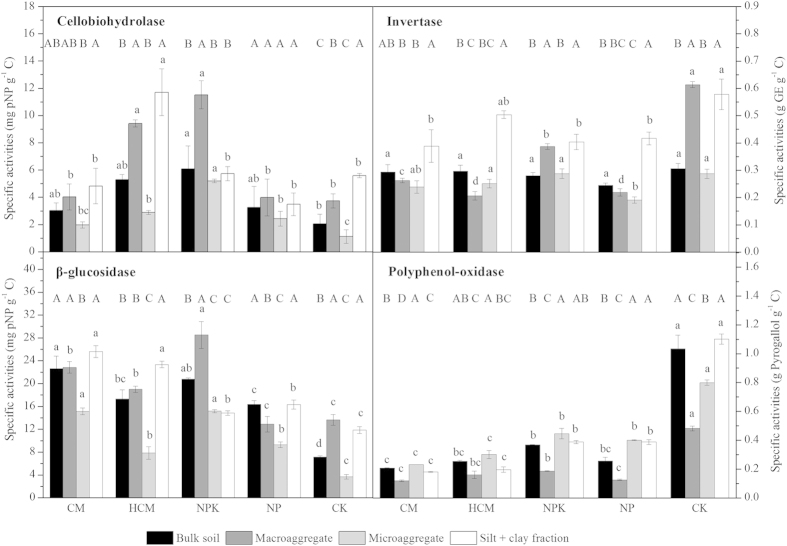
Specific activities of cellobiohydrolase, β-glucosidase, invertase (activity divided by the sum of O-alkyl C and di-O-alkyl C contents) and polyphenol-oxidase (activity divided by the sum of methoxyl/N-alkyl C and phenolic C contents) in bulk soils and aggregates. Different lowercase letters indicate significant differences between treatments for the same aggregate, and different capital letters indicate significant differences between aggregates for the same treatment (Tukey’s test, *P* < 0.05, n = 4).

The specific enzyme activities (enzyme activities per unit of C functional groups) and particle sizes were analyzed to disclose the stability of C functional groups and the “saturation” degree of the mineral particles in bulk soils and aggregates, which helped to explain the possible accumulation mechanism of C functional groups in aggregates. The specific activities of cellobiohydrolase (CBH), invertase and β-glucosidase (BG) in microaggregates were not higher than those in bulk soils, macroaggregates and the silt + clay fractions in all treatments except the specific activities of invertase in HCM and NPK treatments and BG in NPK treatment (Fig. 4). However, the specific activity of polyphenol-oxidase (PPO) in microaggregates was higher than those in bulk soils, macroaggregates and the silt + clay fractions in all treatments except CK. In compost-amended soils, the specific activities of invertase in macroaggregates were also lower than those in bulk soils and the silt + clay fractions. Long-term application of compost increased the specific activities of CBH and BG in bulk soils and aggregates except CBH in the silt + clay fractions, while it decreased that of invertase in macroaggregates and the silt + clay fractions and PPO in bulk soils and aggregates. Compared with compost, the application of NPK mineral fertilizers more effectively increased the specific activities of CBH in bulk soils, macroaggregates and microaggregates and BG in macroaggregates (Fig. 4). According to the results of particle size analysis (Tables S4), clay/OC, silt_20μm_/OC and (clay+silt_20μm_)/OC ratios in aggregates were calculated (Tables S5). All ratios in the silt + clay fractions were largely higher than those in macroaggregates and microaggregates. Except the ratios of silt_20μm_/OC and (clay+silt_20μm_)/OC in microaggregates and clay/OC in the silt + clay fractions, long-term application of compost and mineral fertilizers decreased all ratios in aggregates.

### Correlation and path analysis

The contents of C functional groups were all significantly correlated with the ratio of clay/OC, silt_20μm_/OC or (clay+silt_20μm_)/OC in the silt + clay fractions (*P* < 0.05, Table 1). Compared with the other C functional groups, the content of phenolic C or di-O-alkyl C was more strongly correlated with the silt_20μm_/OC ratio than with the clay/OC ratio. When macroaggregates and microaggregates were further separated into coarse/fine intra particulate organic matter (iPOM) and silt + clay subfractions^20^, significant relationships were found between methoxyl/N-alkyl C content and OC content in coarse iPOM or the silt + clay subfraction, between phenolic C content and the OC content in coarse iPOM, and between carboxyl C content and the OC content in the silt + clay subfraction in macroaggregates (*P* < 0.05, Table 2). By contrast, a significant correlation between the methoxyl/N-alkyl C or phenolic C content and the OC content in the silt + clay subfraction was only observed in microaggregates (*P* < 0.05). The carboxyl C content was found to be marginally correlated with the OC content in the fine iPOM and the silt + clay subfraction in microaggregates (*P* = 0.09 and 0.120, Table 2).

In macroaggregates, the content of OC and C functional groups was not significantly correlated with the mass proportion (Table 3). In microaggregates, the content of OC and all C functional groups, especially methoxyl/N-alkyl C and phenolic C, was significantly negatively correlated with the mass proportion (*P* < 0.05, Table 3). By contrast, the mass proportion in the silt + clay fraction was significantly exponentially related to the content of alkyl C (*P* < 0.05). Correlation analysis indicated that the mass proportion of microaggregates and the silt + clay fractions decreased and macroaggregates mass proportion increased with the sequestration of methoxyl/ N-alkyl C and phenolic C in microaggregates, as well as alkyl C and methoxyl/ N-alkyl C in the silt + clay fractions. Thus, these C functional groups are suggested to be the important binding agents for aggregate formation (Table 3). Path analysis revealed that though *R* square of models from 1 to 4 gradually improved along with the number of variables (the selected C functional groups) increased, only models 1 and 2 had the statistical significance (Table S5). Considering the *P* value of the unstandardized coefficients of variables, only methoxyl/N-alkyl C in microaggregates in the model 1 had the statistical significance. Thus, methoxyl/N-alkyl C in microaggregates was suggested to influence macroaggregation with the direct coefficient of 0.993 and no indirect coefficient was calculated in this study (Table S5).

## Discussion

It is interesting to note that the preferential accumulation of C functional groups was shown to depend on aggregate size, regardless of the type of amendment (fertilizer or compost) input. In the silt + clay fraction, phenolic C and di-O-alkyl C accumulated more quickly than the other C functional groups (Fig. 2). Using the size fractionation method, some studies have demonstrated that phenolic C and polysaccharides could preferentially be preserved in the clay fraction through chemical and biochemical stabilization^11^,^12^, whereas lignin and its phenol products could be protected from further oxidation by silt minerals^4^,^13^. In the current study, enzyme analysis revealed that the reduction in the specific activities of PPO and invertase partly resulted in the accumulation of phenolic C and di-O-alkyl C (Fig. 4). When quantifying the capacity of mineral particles for C sequestration, a ratio of clay/OC of ~10 and a ratio of (clay+silt_20μm_)/OC of ~20 was proposed to represent the “saturation” threshold^26^,^27^. In the present study, the mineral particles in the silt + clay fractions were less saturated and could strongly sequestrate C functional groups (Table S4). Compared with the other C functional groups, the content of phenolic C or di-O-alkyl C was more strongly correlated with the silt_20μm_/OC ratio than with the clay/OC ratio (Table 1), indicating that chemical stabilization and/or biochemical stabilization by fine silt particles (2–20 μm) can be attributed to the preferential accumulation of phenolic C and di-O-alkyl C derived from lignin and polysaccharides.

Carboxyl C was observed to preferentially accumulate in microaggregates compared with the other C functional groups (Fig. 2). Our findings confirm the results of Kinyangi *et al*.^28^ and Wan *et al*.^29^, who found that microaggregates were coated with oxidized carboxyl C, despite the fact that a specific connection mechanism was unclear using X-ray microscopy and near-edge X-ray absorption fine structure spectroscopy. As a heavily oxidized C functional group^12^,^30^,^31^, carboxyl C derived from aliphatic acids, benzene-carboxylic acids, amide and ester structures and quinones can be stabilized by the silt and clay fractions and protected from further oxidation^30^,^32^,^33^. In the present study, the carboxyl C content in microaggregates was marginally (*P* < 0.10) exponentially correlated with the OC content in the fine iPOM subfraction and linearly correlated with that in the silt + clay subfraction (Table 2). These results suggest that the mechanism for the accumulation of carboxyl C in microaggregates might involve the production of carboxyl C from the decomposition of fine iPOM, followed by its stabilization through combining with the silt and clay fractions.

With the high content and heterogeneity of microorganisms and organic matter (OM)^3^,^21^, the turnover of OM in macroaggregates is sensitive to management practices; thus, the easily accumulated OM components within macroaggregates vary greatly in different soils. Compounds containing phenolic and carboxyl C preferentially accumulate in macroaggregates after manure application in a rice-wheat rotation field^6^, whereas O-alkyl and aryl C components preferentially accumulate in macroaggregates in hoop pine-planted soils^34^. In the current study, the levels of phenolic C, carboxyl C and methoxyl/N-alkyl C except carboxyl C in the NP treatment increased more quickly in macroaggregates than those of the other C functional groups (Fig. 2). Phenolic C was mainly located in macroaggregates as coarse iPOM, which is rich in plant-derived SOM (less degraded and fresh SOM) but not in the silt + clay subfraction (Table 2), indicating the accumulated phenolic C in macroaggregates was derived from less degraded/fresh lignin due to its biochemical recalcitrance and/or physical protection^31^,^35^,^36^. Furthermore, the reduced specific activities of PPO in macroaggregates than those in bulk soils in all treatment groups suggest that physical protection from enzymatic degradation by macroaggregates rather than biochemical recalcitrance plays an important role in the accumulation of phenolic C (Fig. 4). Methoxyl/N-alkyl C is mainly located in macroaggregates (as coarse iPOM) and in the silt + clay subfraction (Table 2), indicating that the accumulation of methoxyl/N-alkyl C in macroaggregates might depend on the abundance of less degraded/fresh lignin and lignin derivatives and their chemical stabilization with mineral particles^36^. Carboxyl C was sequestrated in macroaggregates that were mainly distributed in the silt + clay subfraction, which also indicates that they formed rapidly from POM degradation and were then stabilization by mineral particles (Table 2).

The inherent accumulation pattern of C functional groups in aggregates appeared to be only slightly altered by fertilization in arable soils with low OC. Thus, we suggest that the accumulation pattern of SOM was less influenced by the quantity and quality of input organic matters and their decomposition rates, but it instead depended on the nature and characteristics of aggregates, such as mineral particle composition, pore size distribution and so on.

Despite the fact that there was an inherent preferential accumulation of different C functional groups in aggregates, the accumulation pattern of C functional groups in bulk soils was greatly influenced by repeated application of compost and mineral fertilizer due to the changes in increased C functional group contents in aggregates and aggregate mass distribution (Figs 1 and 2). Our results indicate that the widely used method of combining spectroscopic approaches with physical fractionation to elucidate the accumulation of OC components might lead to some biased conclusions because this method neglects the changes in physical fraction mass^6^,^7^,^34^.

In the present study, the accumulation of methoxyl/N-alkyl C, phenolic C, O-alkyl C, di-O-alkyl C in compost-amended soils was mainly distributed in macroaggregates (Fig. 3), despite the fact that the highest increase in the contents of these C functional groups was found in the silt + clay fractions (Fig. 2). Apparently, macroaggregate formation plays a more important role in protecting these C functional groups from decomposition in compost-amended soil compared to CK and NPK (Fig. 1), as found in previous studies^16^,^37^. As discussed above, the lower specific activities of PPO observed in macroaggregates than those in bulk soils in all treatment groups clearly demonstrate that physically protected lignin and/or its derivatives (methoxyl/N-alkyl C and phenolic C) exist in the soils examined in our study^18^,^38^. However, unexpectedly, we did not observe a significant reduction in the specific activity of PPO in compost-amended soil (with higher degrees of macroaggregation) compared with mineral fertilizer-amended soil (Fig. 4). A possible explanation for this result is that the inhibition of PPO activity by N might be as strong as the physical protection of this enzyme by macroaggregation^39^,^40^. Another possibility is that the role of PPO in lignin degradation in soils might not be as important as we had assumed because its redox potential is lower than those of lignin peroxidase and manganese peroxidase, two effective lignin-degrading enzymes^41^.

Agreed with our hypothesis, compost application promoted the accumulation of O-alkyl C and di-O-alkyl C, primarily in macroaggregates. The significant exponential correlation between the content of O-alkyl C or di-O-alkyl C and OC in fine iPOM in macroaggregates (Table 2) confirmed the notion that labile OM encapsulated within macroaggregates in compost-amended soils includes polysaccharides and/or non-cellulosic carbohydrate, as predicted in some previous studies^1^,^6^,^15^,^42^. Furthermore, the specific activities of invertase in macroaggregates were significantly reduced by compost application compared with NPK and CK, and the specific activities of CBH and BG in macroaggregates were also reduced by compost application compared with NPK but not CK (Fig. 4). These results reveal that non-cellulosic carbohydrate accumulation was dominant in macroaggregates in compost-amended soil, and they explain why cellulosic polysaccharides are preferentially decomposed in compost-amended soil compared to non-cellulosic polysaccharides, as observed by Leifeld *et al*.^43^. Unlike methoxyl/N-alkyl C, phenolic C, O-alkyl C and di-O-alkyl C, carboxyl C mainly accumulated in microaggregates in compost-amended soil due to its greater preferential sequestration compared with NPK, NP and CK (Figs 2 and 3). This result can be attributed to the greater production of carboxyl C from the decomposition of fine iPOM and partly to the fact that it is directly derived from compost, which is rich in carboxyl groups^44^.

In the present study, only recalcitrant C functional groups such as alkyl C and methoxyl C were enriched in mineral fertilizer-amended soils. Previous studies have demonstrated that compared with manure, the application of mineral fertilizer more effectively stimulates the decomposition of native SOC and even lignin^12^,^42^. The higher levels of alkyl C were mainly observed in microaggregates, followed by the silt +clay fraction, while methoxyl/N-alkyl C was mainly distributed in macroaggregates, followed by the silt + clay fraction or microaggregates (Fig. 3). The increase of alkyl C and methoxyl C in mineral fertilizer-amended soils was mainly controlled by their accumulation pattern in aggregates but not aggregation.

Disagreed with our hypothesis, organic components characterized by alkyl C and methoxyl/N-alkyl C in the silt + clay fraction and methoxyl/N-alkyl C and phenolic C in microaggregates were found to drive the formation of larger aggregates (Table 3). All of these C functional groups are derived from resistant organic components, except for N-alkyl C, which originates from amino acids and peptides^11^. Path analysis further demonstrated that only methoxyl/N-alkyl C in microaggregates had a direct influence on macroaggregation (Table S5). Although N-alkyl C can associate with Fe oxides through N atoms with lone pair(s) of electrons to form microaggregates^11^,^45^, their labile nature determines that they would not control macroaggregation in arable soil. Methoxyl substituents derived from syringyl and guaiacyl (sinapyl) units of lignin can reduce the mobility of nearby water molecules, producing a hydrophobic effect^31^,^46^. Hydrophobic materials have long-lasting effects on the stability of aggregates because they can accumulate on the surfaces of aggregates and act as cementation agents^47^. Therefore, we confirmed that lignin is mostly associated with macroaggregation^48^, and the accumulation of methoxyl C in microaggregates, which was dominant in aggregate mass in our test soils, could efficiently promote the formation of macroaggregates through the hydrophobic effect.

In the present study, the methoxyl/N-alkyl C content was significantly correlated with the clay/OC, silt_20μm_/OC and (clay+silt_20μm_)/OC ratios in the silt + clay fraction as well as the OC content in the silt + clay subfraction, but not in fine iPOM within microaggregates (Tables 1 and 2). These results imply that methoxyl/N-alkyl C derived from compost is biochemically protected by mineral particles, which in turn promotes macroaggregation. Polysaccharides and phenolic C, preferentially accumulated OC components in the silt + clay fraction, may act as temporary and transient binding agents, as they contain sticky carboxyl (COOH) and carbonyl (C=O) groups, making these compounds water soluble (i.e., polar) and thus, easily decomposable^49^.

**Figure 5:**
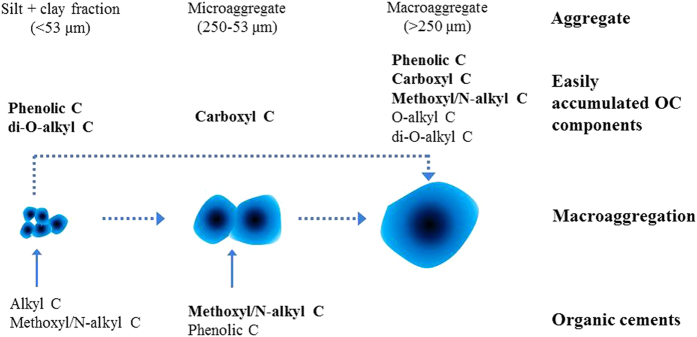
Schematic overview of macroaggregation and OC accumulation mechanisms in aggregates. The dotted arrows indicate the formation of macroaggregate and the solid arrows show the organic cements in microaggregates and the silt + clay fractions.

In conclusion, macroaggregation play a key role in OC components accumulation in our test soils and the accumulation mechanisms could be summarized in Fig. 5. The easily accumulated OC components in aggregate depended on the nature and characteristics of aggregates and were not organic cements. The resistant C functional group methoxyl C in microaggregates could effectively promote macroaggregation; the latter in turn physically and/or biochemically protected methoxyl/N-alkyl C, phenolic C, carboxyl C, O-alkyl C and di-O-alkyl C from biodegradation in macroaggregates by microorganisms.

## Methods

### Field experiment

The long-term field experiment was established in September 1989 in a well-drained field where wheat (*Triticum aestivum* L.) was grown in the winter and maize (*Zea mays* L.) was cultivated in the summer. The site is located in the Fengqiu State Key Agro-Ecological Experimental Station, Fengqiu County, Henan province, China (35°00′N, 114°24′E), a region typical of the North China Plain. The 30-year mean annual air temperature is 13.9 °C and the lowest and highest mean monthly temperatures are −1.0 °C in January and 27.2 °C in July. The mean precipitation is 615 mm, two-thirds of which falls between June and September. The soil is classified as aquic inceptisol, and it has a sandy loam texture and an average pH_H2O_ of 8.2.

A randomized block design was used to prepare four replicates for each of five treatments: compost alone (CM), half compost plus half N fertilizer (HCM), NPK fertilizer (NPK), NP fertilizer (NP) and a no fertilizer control (CK). Each plot measured 9.5 × 5 m^2^. Calcium superphosphate (75 kg P_2_O_5_ ha^−1^ for NPK and NP), potassium sulfate (150 kg K_2_O ha^−1^ for NPK) and compost (1,164 kg C ha^−1^ and 150 kg N ha^−1^ for CM; 582 kg C ha^−1^ and 75 kg N ha^−1^ for HCM) were applied each crop as basal fertilizers. Urea, totaling 150 kg N ha^−1^, was added in two applications: 60 kg N ha^−1^ as basal fertilizer and 90 kg N ha^−1^ as supplemental fertilizer for maize, and 90 kg N ha^−1^ as basal fertilizer and 60 kg N ha^−1^ as supplemental fertilizer for wheat, in the NPK and NP treatments. In the HCM treatment, urea was applied at a rate of 75 kg N ha^−1^ as supplemental fertilizer for maize and 15 kg N ha^−1^ as basal fertilizer and 60 kg N ha^−1^ as supplemental fertilizer for wheat. The insufficient phosphorus and potassium were supplemented with calcium superphosphate and potassium sulfate. No fertilizer or compost was applied in the CK treatment.

### Soil sampling and aggregate fractionation

In October 2007, immediately after the maize harvest, five soil samples were collected in the 0–20 cm soil layer randomly at different locations in each plot using a stainless steel soil sampler with a diameter of 2.5 cm. All samples from each plot were carefully mixed to form a composite and immediately transported to the laboratory for analysis. Moist soils were gently broken apart along the natural break points and passed through an 8-mm sieve. Visible plants and organic debris (which were not incorporated into aggregates) that passed through the sieve were carefully removed by hand. After thorough mixing, a subsample was dried at 105 °C for soil moisture measurements. Another subsample was air-dried and used for soil property analysis. The remaining field-moist soil was used for wet-sieving according to the protocol of Elliott^50^. The tested soil samples were divided into macroaggregates (>250 μm), microaggregates (53–250 μm) and the silt + clay fraction (<53 μm). Organic C contents in soil and aggregates were determined by the wet oxidation-redox titration method^51^. Particle sizes in soil and aggregates were determined using the pipette method^52^. Combined with the application of particle size and density fractionation method, macroaggregates and microaggregates were further fractionated into different subfractions including coarse iPOM, fine iPOM, and silt + clay subfractions. The detailed description of the fractionation method and the results were reported in our previous paper^24^.

### NMR spectroscopy

Solid-state cross-polarization magic-angle spinning (CPMAS) ^13^C NMR spectroscopy of organic matter in bulk soils and aggregates was performed. To remove paramagnetic compounds and increase the organic C contents, soil samples were treated with hydrofluoric acid (HF) solution prior to ^13^C NMR spectroscopy. For each sample, the successive treatment involved shaking 5 g of soil in 50 mL of 10% HF solution for periods of 1 h (four times), 12 h (three times) and 24 h (once), respectively^53^. Between treatments, the samples were centrifuged for 10 min at 1,680 *g* and the supernatant was discarded and replaced with fresh 10% HF solution. After the final treatment, the soil sample was washed with deionized water to pH 6–7 and then freeze-dried. ^13^C NMR analyses were performed on a Bruker Avance III 400 spectrophotometer at 100.6  MHz (400.13 MHz ^1^H frequency, Bruker BioSpin Corporation, Switzerland). All experiments were run in a double resonance probe head using 7-mm sample rotors. The spectrometer operated at a spinning speed of 6 kHz, contact time of 10 ms, ^1^H 90° pulse length of 4 μs and a recycle delay of 0.5 s. Four-pulse total suppression of sidebands (TOSS) was employed before detection, with two-pulse phase modulated decoupling applied for optimum resolution^54^.

### Calculation and statistical analysis

To facilitate interpretation of the ^13^C NMR spectra, the overall chemical shift range was divided into the following: alkyl-C (0–45 ppm), methoxyl/N-alkyl C (45–60 ppm), O-alkyl C (60–93 ppm), di-O-alkyl C (93–110 ppm), aromatic C (110–142 ppm), phenolic C (142–160 ppm) and carboxyl C (160–190 ppm). Areas of the chemical shift regions were measured by integration and were described as the percentage of total area (relative content). According to Hilscher and Knicker^55^, the relative content was multiplied by the organic C content to calculate the content of C functional groups. The amount of C functional groups in aggregates was calculated by multiplying its content with the proportion of the aggregate mass. Because the results of ^13^C CP/TOSS NMR are semiquantitative, the relative content, content and amount of C functional groups have to be interpreted with care, and only the same C functional group was compared among treatments or aggregates. Similar to the decomposition rate calculated by Hilscher and Knicker^55^, the increase in C functional group content or amount in fertilized soils was calculated compared to the corresponding values in the CK treatment to analyze the accumulation efficiency of different C functional groups. The increase in the proportion of C functional groups in aggregates was calculated by dividing the increased amount of C functional groups in aggregates by the increased content of C functional groups in bulk soil compared with the CK.

According to Schjønning *et al*.^27^, the specific ratios of either clay (<2 μm), silt (2–20 μm) or clay + silt (<20 μm) mass to organic C (OC) content were quantified in bulk soils and aggregates to test the “saturation” degree of the mineral particles. Based on the measurements of Yu *et al*.^18^, the enzyme activity per unit of C functional groups, which is termed the specific activity of the enzyme were quantified. The enzymes includes three carbohydrate hydrolases (CBH, β-glucosidase and invertase) and one important lignin degrading enzyme (polyphenol-oxidase, PPO). Therefore, the specific activity of CBH, β-glucosidase and invertase were divided by the sum of the O-alkyl C and di-O-alkyl C contents, while the specific activity of polyphenol-oxidase (PPO) was divided by the sum of the methoxyl/N-alkyl C and phenolic C contents.

Statistically significant differences among treatments, soils or aggregates were identified using Turkey’s test at *P* = 0.05. Statistical relationships were obtained through regression analyses between the following parameters: (1) the content of C functional groups and the ratio of clay/OC, silt_20μm_/OC or clay+silt_20μm_/OC in the silt + clay fractions; (2) the content of C functional groups and OC content in physical subfractions in macroaggregates and microaggregates cited from Yu *et al*.^24^ and (3) the content of C functional groups and aggregate mass proportion. Based on the results of regression analyses of (3), the C functional groups with higher *R*^2^ than SOC were selected as independent variables and path analysis was conducted to quantify the roles of these C functional groups in macroaggregation (mass proportion of macroaggregates as dependent variable). Before analysis, all data (including independent variables and dependent variable) were checked for normality and transformed using natural logarithms. Path coefficients (direct effects) were obtained through stepwise regression and then indirect effects were calculated by the path coefficients multiplied by the correlation coefficients between corresponding variables. The statistical analyses were performed with SPSS 13.0 software in windows XP ((IBM corporation, Chicago, IL, USA).

## Additional Information

**How to cite this article**: Yu, H. *et al*. Accumulation of organic C components in soil and aggregates. *Sci. Rep*. **5**, 13804; doi: 10.1038/srep13804 (2015).

## Supplementary Material

Supplementary Information

## Figures and Tables

**Table 1:** Relationship between C functional group contents (*y*) and clay/OC, silt_20μm_/OC and (clay+silt_20μm_)/OC ratios in the silt + clay fraction (*x*).

C functional groups	Clay/OC ratio		Silt_20_ _μm_/OC ratio		(Clay+silt_20μm_)/OC ratio	
	Equation	*R*^2^	Equation	*R*^2^	Equation	*R*^2^
Alkyl C	*y* = −1.174ln*x*+6.183	0.981**	*y *= −0.752ln*x*+4.284	0.853*	*y* = −0.946ln*x*+5.814	0.931**
Methxoyl/N-alkyl C	*y* = −0.753ln*x*+3.903	0.947**	*y* = −0.519ln*x*+2.844	0.954**	*y* = −0.634ln*x*+3.806	0.983**
O-alkyl C	*y* = −1.772ln*x*+9.389	0.974**	*y* = −1.204ln*x*+6.819	0.952**	*y* = −1.481ln*x*+9.103	0.995**
di-O-alkyl C	*y *= −0.603ln*x*+3.149	0.879*	*y* = −0.439ln*x*+2.402	0.990**	*y*= − 0.526ln*x*+3.162	0.979**
Aromatic C	*y* = −1.137ln*x*+5.959	0.953**	*y* = −0.781ln*x*+4.348	0.954**	*y* = −0.957ln*x*+5.807	0.986**
Phenolic C	*y* = −0.463ln*x*+2.339	0.939**	*y* = −0.324ln*x*+1.710	0.978**	*y* = −0.394ln*x*+2.301	0.996**
Carboxyl C	*y* = −1.248ln*x*+6.405	0.961**	*y* = −0.858ln*x*+4.639	0.963**	*y* = −1.051ln*x*+6.242	0.996**

*P < 0.05; **P < 0.01.

**Table 2:** Relationship between C functional group contents (*y*) and C contents in physical subfractions (*x*) in macroaggregates and microaggregates.^a^

Subfraction	C functional groups	Macroaggregates (>250 μm)		Microaggregates (53–250 μm)	
		Equation	*R*^2^	Equation	*R*^2^
Coarse iPOM	Alkyl C	*y* = 10.206e^0.305*x*^	0.743	–	–
	Methxoyl/N-alkyl C	*y* = 1.961ln*x*−4.393	0.865*	–	–
	O-alkyl C	*y* = 10.096e^0.137*x*^	0.599	–	–
	di-O-alkyl C	*y* = 10.091e^0.388*x*^	0.612	–	–
	Aromatic C	*y* = 8.199e^0.289*x*^	0.585	–	–
	Phenolic C	*y* = 1.752ln*x*−4.044	0.872*	–	–
	Carboxyl C	*y* = 2.837ln*x*−6.449	0.687	–	–
Fine iPOM	Alkyl C	*y* = 1.562e^0.766*x*^	0.399	*y* = 2.151*x*+2.749	0.197
	Methxoyl/N-alkyl C	*y* = 0.514ln*x*+0.206	0.696	*y* = 2.686ln*x*+6.183	0.396
	O-alkyl C	*y* = 0.797e^0.555*x*^	0.837*	*y*=3.684ln*x*+2.891	0.600
	di-O-alkyl C	*y* = 0.836e^1.526*x*^	0.807*	*y* = 3.585ln*x*+6.877	0.632
	Aromatic C	*y* = 0.304e^1.226*x*^	0.898*	*y* = 2.625*x*+1.526	0.364
	Phenolic C	*y* = 0.419ln*x*+0.125	0.585	*y* = 2.939ln*x*+7.314	0.520
	Carboxyl C	*y* = 0.194ln*x*+0.406	0.534	*y* = 0.258e^0.242*x*^	0.670
Silt + clay subfraction	Alkyl C	*y* = 1.101e^1.318*x*^	0.848*	*y* = 3.463*x*+4.559	0.513
	Methxoyl/N-alkyl C	*y* = 0.46ln*x*+0.105	0.780*	*y* = 6.231*x*+3.911	0.838*
	O-alkyl C	*y* = 1.08e^0.584*x*^	0.663	*y* = 2.350*x*+3.660	0.688
	di-O-alkyl C	*y* = 1.24e^1.522*x*^	0.575	*y* = 7.161*x*+3.585	0.785
	Aromatic C	*y* = 0.471e^1.205*x*^	0.621	*y* = 4.415e^0.445*x*^	0.650
	Phenolic C	*y* = 0.398ln*x*+0.002	0.735	*y* = 8.429*x*+4.012	0.847*
	Carboxyl C	*y* = 0.776ln*x*-0.145	0.842*	*y* = 0.187*x*-0.564	0.609

^a^The data for C content in physical subfractions are cited from Yu *et al*.^20^, and iPOM is the abbreviation for intra particulate organic matter. *P < 0.05; **P < 0.01.

**Table 3:** Correlation between the C functional group contents and aggregate mass ratios in macroaggregates, microaggregates and the silt + clay fraction.

C function group	Macroaggregates(>250 μm)		Microaggregates (53-250 μm)		Silt + clay fraction (<53 μm)	
	Equation	*R*^2^	Equation	*R*^2^	Equation	*R*^2^
SOC	–	0.301	*y* = −3.283*x*+85.620	0.943**	*y* = 25.638e^−0.049*x*^	0.714
Alkyl C	–	0.330	*y* = −18.451*x*+81.857	0.767	*y* = 26.579e^−0.320*x*^	0.807*
O-alkyl C	–	0.247	*y* = −7.153*x*+85.505	0.946**	*y* = 25.626e^−0.108*x*^	0.684
* Methoxyl/N-alkyl C*	–	0.318	*y* = −30.787*x*+83.477	0.984**	*y* = 25.298e^−0.467*x*^	0.732
* O-alkyl C*	–	0.177	*y* = −12.487*x*+86.348	0.906*	*y*=26.079e^−0.193*x*^	0.672
* di-O-alkyl C*	–	0.187	*y* = −35.879*x*+85.712	0.939**	*y* = 24.825e^−0.521*x*^	0.628
Aryl C	–	0.251	*y* = −13.371*x*+86.746	0.917*	*y* = 25.133e^−0.215*x*^	0.695
* Aromatic C*	–	0.157	*y* = −17.570*x*+86.002	0.873*	*y* = 25.700e^−0.306*x*^	0.710
* Phenolic C*	–	0.611	*y* = −39.857*x*+83.157	0.983**	*y* = 23.820e^−0.717*x*^	0.658
Carboxyl C	–	0.307	*y* = −16.925*x*+79.837	0.842*	*y* = 24.560e^−0.271*x*^	0.667

*P* < 0.05; **P < 0.01.

## References

[b1] Ngo P., Rumpel C., Doan T. & Jouquet P. The effect of earthworms on carbon storage and soil organic matter composition in tropical soil amended with compost and vermicompost. Soil Biol. Biochem. 50, 214–220 (2012).

[b2] Bolan N. S., Kunhikrishnan A. & Naidu R. Carbon storage in a heavy clay soil landfill site after biosolid application. Sci. Total Environ. 465, 216–225 (2013). 23380138 10.1016/j.scitotenv.2012.12.093

[b3] Thangarajan R., Bolan N. S., Tian G., Naidu R. & Kunhikrishnan A. Role of organic amendment application on greenhouse gas emission from soil. Sci. Total Environ. 465, 72–96 (2013). 23433468 10.1016/j.scitotenv.2013.01.031

[b4] Heim A. & Schmidt M. W. I. Lignin is preserved in the fine silt fraction of an arable Luvisol. Org. Geochemi. 38, 2001–2011 (2007).

[b5] Six J. & Paustian K. Aggregate-associated soil organic matter as an ecosystem property and a measurement tool. Soil Biol. Biochem. 68, 4–9 (2014).

[b6] Wang Q. . The effects of compost in a rice-wheat cropping system on aggregate size, carbon and nitrogen content of the size-density fraction and chemical composition of soil organic matter, as shown by ^13^C CP NMR spectroscopy. Soil Use Manage. 28, 337–346 (2012).

[b7] Ryals R., Kaiser M., Torn M. S., Berhe A. A. & Silver W. L. Impacts of organic matter amendments on carbon and nitrogen dynamics in grassland soils. Soil Biol. Biochem. 68, 52–61 (2014).

[b8] Wagai R., Mayer L. M. & Kitayama K. Nature of the “occluded” low-density fraction in soil organic matter studies: a critical review. Soil Sci. Plant Nutr. 55, 13–25 (2009).

[b9] Courtier-Murias D. . Unraveling the long-term stabilization mechanisms of organic materials in soils by physical fractionation and NMR spectroscopy. Agr. Ecosyst. Environ. 171, 9–18 (2013).

[b10] Jindaluang W., Kheoruenromne I., Suddhiprakarn A., Singh B. P. & Singh B. Influence of soil texture and mineralogy on organic matter content and composition in physically separated fractions soils of Thailand. Geoderma 195-196, 207–219 (2013).

[b11] Schöning I., Morgenroth G. & Kögel-Knabner I. O/N-alkyl C and alkyl C are stabilised in fine particle size fractions of forest soils. Biogeochemistry 73, 475–497 (2005).

[b12] Nobili M. De., Contin M., Mahieu N., Randall E. W. & Brookes P. C. Assessment of chemical and biochemical stabilization of organic C in soils from the long-term experiments at Rothamsted (UK). Waste Manage. 28, 723–733 (2008). 10.1016/j.wasman.2007.09.025 18042372

[b13] Clemente J. S. Soil organic matter composition impacts its degradability and association with soil minerals. A PhD dissertation of University of Toronto, Toronto, Canada (2012).

[b14] He Y., Chen C. R., Xu Z. H., Williams D. & Xu J. M. Assessing management impacts on soil organic matter quality in subtropical Australian forests using physical and chemical fractionation as well as ^13^C NMR spectroscopy. Soil Biol. Biochem. 41, 640–650 (2009).

[b15] Grandy A. S., Porter G. A. & Erich M. S. Organic amendment and rotation crop effects on the recovery of soil organic matter and aggregation in potato cropping systems. Soil Sci. Soc. Am. J. 66, 1311–1319 (2002).

[b16] Bipfubusa M., Angers D. A., N’Dayegamiye A. & Antoun H. Soil aggregation and biochemical properties following the application of fresh and composted organic amendments. Soil Sci. Soc. Am. J. 72, 160–166 (2008).

[b17] Simonetti G. . Characterization of humic carbon in soil aggregates in a long-term experiment with manure and mineral fertilization. Soil Sci. Soc. Am. J. 76, 880–890 (2011).

[b18] Yu H. Y., Ding W. X., Luo J. F., Donnision A. & Zhang J. B. Long-term effect of compost and inorganic fertilizer on activities of carbon-cycle enzymes in aggregates of an intensively cultivated sandy loam. Soil Use Manage. 28, 347–360 (2012a).

[b19] Kimetu J. M. & Lehmann J. Stability and stabilisation of biochar and green manure in soil with different organic carbon contents. Aust. J. Soil Res. 48, 577–585 (2010).

[b20] von Lützowa M. . SOM fractionation methods: relevance to functional pools and to stabilization mechanisms. Soil Biol. Biochem. 39, 2183–2207 (2007).

[b21] Aoyama M., Angers D. A., N’Dayegamiye A. & Bissonnette N. Protected organic matter in water-stable aggregates as affected by mineral fertilizer and manure applications. Can. J. Soil Sci. 79, 419–425 (1999).

[b22] Mikha M. M. & Rice C. W. Tillage and manure effects on soil and aggregate-associated carbon and nitrogen. Soil Sci. Soc. Am. J. 68, 809–816 (2004).

[b23] Hai L., Li X. G., Li F. M., Suo D. R. & Guggenberger G. Long-term fertilization and manuring effects on physically-separated soil organic matter pools under a wheat-wheat-maize cropping system in an arid region of China. Soil Biol. Biochem. 42, 253–259 (2010).

[b24] Yu H. Y., Ding W. X., Luo J. F., Geng R. L. & Cai Z. C. Long-term application of organic manure and mineral fertilizers on aggregation and aggregate-associated carbon in a sandy loam soil. Soil Till. Res. 124, 170–177 (2012b).

[b25] Abiven S., Menasse S., Angers D. A. & Leterme P. A model to predict soil aggregate stability dynamics following organic residue incorporation under field conditions. Soil Soil Sci. Soc. Am. J. 72, 119–125 (2008).

[b26] Dexter A. R. . Complexed organic matter controls soil physical properties. Geoderma 144, 620–627 (2008).

[b27] Schjønning P., de Jonge L. W., Moldrup P., Christensen B. T. & Olesen J. E. Searching the critical soil organic carbon threshold for satisfactory tilth conditions - test of the Dexter clay:carbon hypothesis. (eds de Jonge L. W., Moldrup P. & Vendelboe A. L.) 341–346 (Proceedings of the 1st International Conference and Exploratory Workshop on Soil Architecture and Physico-chemical Functions “Cesar”, Tjele, Denmark, 2010).

[b28] Kinyangi J. . Nanoscale biogeocomplexity of the organomineral assemblage in soil: application of STXM microscopy and C 1s-NEXAFS spectroscopy. Soil Science Soil Sci. Soc. Am. J. 70, 1708–1718 (2006).

[b29] Wan J., Tyliszczak T. & Tokunaga T. K. Organic carbon distribution, speciation, and elemental correlations within soil microaggregates: applications of STXM and NEXAFS spectroscopy. Geochim. Cosmochim. Ac. 71, 5439–5449 (2007).

[b30] Mahieu M., Powlson D. S. & Randall E. W. Statistical analysis of published carbon-13 CPMAS NMR spectra of soil organic matter. Sci. Soc. Am. J. 63, 307–319 (1999).

[b31] Conte P., Spaccini R., Chiarella M. & Piccolo A. Chemical properties of humic substances in soils of an Italian volcanic system. Geoderma 117, 243–250 (2003).

[b32] Helfrich M., Ludwig B., Buurman P. & Flessa H. Effect of land use on the composition of soil organic matter in density and aggregate fractions as revealed by solid-state 13C NMR spectroscopy. Geoderma 136, 331–341 (2006).

[b33] Dippold M. A. & Kuzyakov Y. Biogeochemical transformations of amino acids in soil assessed by position specific labeling. Plant Soil 373, 385–401 (2013).

[b34] He Y., Chen C. R., Xu Z. H., Williams D. & Xu J. M. Assessing management impacts on soil organic matter quality in subtropical Australian forests using physical and chemical fractionation as well as ^13^C NMR spectroscopy. Soil Biol. Biochem. 41, 640–650 (2009).

[b35] Mikutta R., Kleber M., Torn M. S. & Jahn R. Stabilization of soil organic matter: association with minerals or chemical recalcitrance? Biogeochemistry 77, 25–56 (2006).

[b36] Zhou P., Pan G. X., Spaccini R. & Piccolo A. Molecular changes in particulate organic matter (POM) in a typical Chinese paddy soil under different long-term fertilizer treatments. Eur. J. Soil Sci. 61, 231–242 (2010).

[b37] Angers D. A. & Giroux M. Recently deposited organic matter in soil water-stable aggregates. Sci. Soc. Am. J. 60, 1547–1551 (1996).

[b38] Pulleman M. M., Six J., van Breemen N. & Jongmans A. G. Soil organic matter distribution and microaggregate characteristics as affected by agricultural management and earthworm activity. Eur. J. Soil Sci. 56, 453–467 (2005).

[b39] Frey S. D., Knorr M., Parrent J. L. & Simpson R. T. Chronic nitrogen enrichment affects the structure and function of the soil microbial community in temperate hardwood and pine forests. For. Ecol. Manage. 196, 159–171 (2004).

[b40] Chalhoub M., Garnier P., Coquet Y., Mary B. & Lafolie F. Increased nitrogen availability in soil after repeated compost applications: use of the PASTIS model to separate short and long-term effects. Soil Biol. Biochem. 65, 144–157 (2013).

[b41] Fujii K., Uemura M., Hayakawa C., Funakawa S. & Kosaki T. Environmental control of lignin peroxidase, manganese peroxidase and laccase activities in forest floor layers in humid Asia. Soil Biol. Biochem. 57, 109–115 (2013).

[b42] Moreno-Cornejo J., Zornoza R., Faz A. & Rosales R. M. Effects of pepper crop residues and inorganic fertilizers on soil properties relevant to carbon cycling and broccoli production. Soil Use Manage. 29, 519–530 (2013).

[b43] Leifeld J., Siebert S. & Kögel-Knabner I. Changes in the chemical composition of soil organic matter after application of compost. Eur. J. Soil Sci. 53, 299–309 (2002).

[b44] Ellerbrock R. H., Höhn A. & Rogasik J. Functional analysis of soil organic matter as affected by long-term manorial treatment. Eur. J. Soil Sci. 50, 65–71 (1999).

[b45] Deng Y. & Dixon J. B. (Soil organic matter and organic–mineral interactions.) Soil Mineralogy with Environmental Applications (eds Dixon J. B. & Schulze D. G.) 69–107 (SSSA Book Ser. No. 7. SSSA, Madison, WI, USA, 2002).

[b46] Vu T., Chaffee A. & Yarovsky I. Investigation of lignin-water interactions by molecular simulation. Mol. Simulat. 28, 981–991 (2002).

[b47] Lal R. Soil carbon management and climate change. Carbon Manage. 4, 439–462 (2014).

[b48] Monreal C. M., Schulten H.-R. & Kodama H. Age, turnover and molecular diversity of soil organic matter in aggregates of a Gleysol. Can. J. Soil Sci. 77, 379–388 (1997).

[b49] Nichols K. & Halvorson J. J. Roles of biology, chemistry, and physics in soil macroaggregate formation and stabilization. Open Agr. J. 7, 107–117 (2013).

[b50] Elliott E. T. Aggregate structure and carbon, nitrogen, and phosphorus in native and cultivated soils. Sci. Soc. Am. J. 50, 627–633 (1986).

[b51] Carter M. R. Soil sampling and methods of analysis. 190–191 (Lewis Publishers, Boca Raton, 1993).

[b52] Buchan G. D., Grewal K. S., Claydon J. J. & Mcpherson R. J. A comparison of sedigraph and pipette methods for soil particle-size analysis. Aust. J. Soil Res. 31, 407–417 (1993).

[b53] Zhuang S. Y., Sun X., Liu G. Q., Wong M. H. & Cao Z. H. Carbon sequestration in bamboo plantation soil with heavy winter organic mulching management. Bot. Rev. 77, 252–261 (2011).

[b54] Cao X. Y. . Solid-state NMR analysis of soil organic matter fractions from integrated physical-chemical extraction. Soil Sci. Soc. Am. J. 75, 1374–1384 (2011).

[b55] Hilscher A. & Knicker H. Carbon and nitrogen degradation on molecular scale of grass-derived pyrogenic organic material during 28 months of incubation in soil. Soil Biol. Biochem. 43, 261–270 (2011).

