# Supplementary material for: Accumulation of organic C components in soil and aggregates
Source: Sci Rep. 2015 Sep 11;5:13804. doi: 10.1038/srep13804 (PMC4566104; doi:10.1038/srep13804)
Supplement: Supplementary Information [file srep13804-s1.doc]

**Title:** Accumulation of organic C components in soil and aggregates

**Authors:** Hongyan Yu^1,2^, Weixin Ding^1,*^, Zengming Chen^1^, Huanjun Zhang^1^, Jiafa Luo^3^, Nanthi Bolan^4^

**Affiliations:**

^1^ State Key Laboratory of Soil and Sustainable Agriculture, Institute of Soil Science, Chinese Academy of Sciences, Nanjing 210008, China

^2^ School of Environment and Civil Engineering, Jiangnan University, Wuxi 214122, China

^3^ Land and Environment, AgResearch, Hamilton 3240, New Zealand

^4^ Centre for Environmental Risk Assessment and Remediation, University of South Australia, SA 5095, Australia

**Author for correspondence:** Weixin Ding

State Key Laboratory of Soil and Sustainable Agriculture, Institute of Soil Science, Chinese Academy of Sciences, Nanjing 210008, China

Tel: 0086-25-8688-1527; Fax: 0086-25-8688-1000; Email: [wxding@issas.ac.cn](mailto:wxding@issas.ac.cn)

**Number of figures:** 1

**Number of tables:** 6

**Figure S1.** ^13^C-CPMAS-NMR spectra of organic matter in bulk soils and aggregates, as affected by long-term application of compost and mineral fertilizers.

**Table S1.** Relative content of different C functional groups in bulk soils and aggregates as affected by long-term application of compost and fertilizers.

| Aggregate | Treatment | Alkyl C (%) | O-alkyl C (%) | | | Aryl C (%) | | Carboxyl C (%) |
| --- | --- | --- | --- | --- | --- | --- | --- | --- |
|  |  |  | Methoxyl/N-alkyl C | O-alkyl C | di-O-alkyl C | Aromatic C | Phenolic C |  |
| Bulk soil | CM | 13.10 (1.42) | 9.80 (0.88) | 26.00 (2.28) | 9.40 (0.51) | 18.50 (0.96) | 8.00 (0.36) | 15.20 (1.27) |
|  | HCM | 17.20 (1.77) | 9.80 (0.79) | 29.30 (2.83) | 8.10 (0.65) | 15.70 (1.10) | 5.90 (0.29) | 14.00 (1.66) |
|  | NPK | 17.70 (2.01) | 11.20 (1.61) | 27.90 (1.30) | 8.20 (0.65) | 16.30 (1.06) | 5.70 (0.22) | 13.00 (2.73) |
|  | NP | 19.38 (2.93) | 9.69 (0.94) | 24.81 (2.10) | 8.53 (1.14) | 17.83 (1.08) | 5.62 (0.56) | 14.15 (3.40) |
|  | CK | 16.10 (1.07) | 8.20 (0.71) | 26.50 (1.56) | 9.00 (0.85) | 20.30 (0.83) | 6.10 (0.29) | 13.80 (1.81) |
| Macroaggregate | CM | 13.53 (1.77) | 9.74 (0.59) | 27.33 (2.25) | 9.88 (0.75) | 18.81 (0.57) | 7.98 (0.66) | 12.72 (2.47) |
|  | HCM | 12.80 (0.65) | 8.58 (1.09) | 29.32 (3.46) | 9.86 (1.08) | 19.46 (1.00) | 6.53 (0.78) | 13.44 (1.21) |
|  | NPK | 13.32 (0.70) | 9.05 (0.78) | 28.83 (0.80) | 9.59 (0.51) | 20.64 (1.23) | 6.39 (0.35) | 12.18 (0.69) |
|  | NP | 10.04 (1.20) | 9.24 (0.84) | 30.72 (2.98) | 11.45 (1.02) | 20.28 (1.09) | 7.63 (0.70) | 10.64 (1.87) |
|  | CK | 13.28 (0.79) | 8.10 (0.36) | 28.42 (0.77) | 10.09 (1.28) | 24.57 (1.97) | 5.71 (0.75) | 9.83 (0.58) |
| Microaggregate | CM | 14.37 (0.87) | 10.34 (1.12) | 26.15 (1.35) | 9.05 (0.64) | 17.67 (1.31) | 7.61 (0.98) | 14.80 (2.06) |
|  | HCM | 13.91 (2.35) | 9.04 (0.75) | 27.54 (0.86) | 8.90 (0.62) | 20.03 (1.25) | 6.12 (1.24) | 14.46 (1.60) |
|  | NPK | 15.92 (2.43) | 8.92 (0.93) | 26.91 (1.24) | 8.60 (0.24) | 17.99 (1.13) | 6.69 (0.25) | 14.97 (0.77) |
|  | NP | 10.75 (1.06) | 9.57 (1.03) | 28.92 (1.01) | 10.32 (1.12) | 19.46 (0.69) | 7.53 (0.07) | 13.44 (1.62) |
|  | CK | 15.90 (2.38) | 9.70 (0.71) | 26.71 (1.52) | 8.59 (0.66) | 22.42 (3.25) | 6.20 (0.91) | 10.49 (0.56) |
| Silt + clay fraction | CM | 17.12 (1.96) | 10.79 (1.01) | 25.86 (2.20) | 8.39 (0.62) | 15.92 (1.18) | 6.16 (0.52) | 15.75 (1.12) |
|  | HCM | 16.56 (0.95) | 9.77 (1.19) | 27.65 (2.51) | 8.77 (0.83) | 16.56 (0.59) | 4.97 (0.48) | 15.73 (1.58) |
|  | NPK | 17.83 (1.24) | 10.52 (0.89) | 27.27 (1.88) | 8.02 (0.61) | 16.40 (0.65) | 4.81 (0.25) | 15.15 (1.00) |
|  | NP | 14.60 (1.85) | 10.22 (1.08) | 28.03 (2.33) | 9.34 (0.93) | 17.37 (1.17) | 5.84 (0.20) | 14.60 (0.93) |
|  | CK | 23.70 (3.47) | 10.90 (1.04) | 27.25 (1.35) | 6.16 (0.52) | 14.45 (0.72) | 3.32 (0.21) | 14.22 (2.11) |

Numbers in parentheses denote the standard error of the means (*n* = 4).

**Table S2.** Content of different C functional groups (g C kg^-1^ aggregate) in bulk soils and aggregates as affected by long-term application of compost and fertilizers.

| Aggregate | Treatment | Alkyl C  (g C kg^-1^) | O-alkyl C (g C kg^-1^) | | | Aryl C (g C kg^-1^) | | Carboxyl C (g C kg^-1^) |
| --- | --- | --- | --- | --- | --- | --- | --- | --- |
|  |  |  | Methoxyl/N-alkyl C | O-alkyl C | di-O-alkyl C | Aromatic C | Phenolic C |  |
| Bulk soil | CM | 1.30 (0.15) | 0.97 (0.09) | 2.58 (0.23) | 0.93 (0.05) | 1.83 (0.10) | 0.79 (0.03) | 1.50 (0.13) |
|  | HCM | 1.31 (0.15) | 0.75 (0.07) | 2.23 (0.21) | 0.62 (0.05) | 1.20 (0.09) | 0.45 (0.02) | 1.08 (0.12) |
|  | NPK | 1.00 (0.10) | 0.63 (0.09) | 1.56 (0.06) | 0.46 (0.04) | 0.92 (0.05) | 0.32 (0.02) | 0.73 (0.15) |
|  | NP | 1.05 (0.16) | 0.53 (0.05) | 1.35 (0.11) | 0.46 (0.06) | 0.97 (0.05) | 0.31 (0.03) | 0.77 (0.18) |
|  | CK | 0.71 (0.05) | 0.36 (0.03) | 1.18 (0.06) | 0.40 (0.04) | 0.90 (0.03) | 0.27 (0.01) | 0.61 (0.07) |
| Macroaggregate | CM | 1.65 (0.21) | 1.19 (0.07) | 3.33 (0.28) | 1.20 (0.08) | 2.29 (0.09) | 0.97 (0.09) | 1.55 (0.29) |
|  | HCM | 1.51 (0.08) | 1.01 (0.13) | 3.47 (0.40) | 1.17 (0.13) | 2.30 (0.12) | 0.77 (0.09) | 1.59 (0.15) |
|  | NPK | 1.56 (0.11) | 1.06 (0.11) | 3.25 (0.03) | 1.12 (0.07) | 2.41 (0.14) | 0.75 (0.04) | 1.54 (0.09) |
|  | NP | 1.10 (0.14) | 1.01 (0.10) | 3.37 (0.31) | 1.26 (0.11) | 2.22 (0.13) | 0.84 (0.08) | 1.17 (0.21) |
|  | CK | 0.88 (0.05) | 0.54 (0.02) | 1.88 (0.04) | 0.67 (0.08) | 1.63 (0.14) | 0.38 (0.05) | 0.65 (0.04) |
| Microaggregate | CM | 1.26 (0.04) | 0.91 (0.09) | 2.30 (0.07) | 0.80 (0.04) | 1.56 (0.09) | 0.67 (0.07) | 1.30 (0.16) |
|  | HCM | 1.06 (0.20) | 0.69 (0.14) | 2.09 (0.42) | 0.68 (0.14) | 1.52 (0.43) | 0.46 (0.21) | 1.10 (0.37) |
|  | NPK | 0.80 (0.13) | 0.45 (0.06) | 1.36 (0.07) | 0.43 (0.01) | 0.91 (0.07) | 0.34 (0.02) | 0.75 (0.02) |
|  | NP | 0.52 (0.05) | 0.46 (0.05) | 1.39 (0.05) | 0.50 (0.06) | 0.94 (0.02) | 0.36 (0.00) | 0.65 (0.07) |
|  | CK | 0.67 (0.10) | 0.41 (0.03) | 1.13 (0.07) | 0.36 (0.02) | 0.95 (0.12) | 0.26 (0.04) | 0.44 (0.03) |
| Silt + clay fraction | CM | 1.80 (0.18) | 1.13 (0.09) | 2.72 (0.18) | 0.88 (0.06) | 1.67 (0.10) | 0.65 (0.04) | 1.66 (0.11) |
|  | HCM | 1.21 (0.08) | 0.72 (0.08) | 2.02 (0.16) | 0.64 (0.06) | 1.21 (0.03) | 0.36 (0.03) | 1.15 (0.11) |
|  | NPK | 1.03 (0.06) | 0.61 (0.05) | 1.57 (0.10) | 0.46 (0.03) | 0.95 (0.05) | 0.28 (0.01) | 0.87 (0.06) |
|  | NP | 0.59 (0.07) | 0.41 (0.04) | 1.13 (0.10) | 0.38 (0.04) | 0.70 (0.05) | 0.23 (0.01) | 0.59 (0.03) |
|  | CK | 0.75 (0.12) | 0.34 (0.03) | 0.86 (0.05) | 0.19 (0.02) | 0.46 (0.03) | 0.10 (0.01) | 0.45 (0.07) |

Numbers in parentheses denote the standard error of the means (*n* = 4).

**Table S3.** Amount of different C functional groups (g C kg^-1^ soil) in aggregates as affected by long-term application of compost and fertilizers.

| Aggregate | Treatment | Alkyl C  (g C kg^-1^) | O-alkyl C (g C kg^-1^) | | | Aryl C (g C kg^-1^) | | Carboxyl C (g C kg^-1^) |
| --- | --- | --- | --- | --- | --- | --- | --- | --- |
|  |  |  | Methoxyl/N-alkyl C | O-alkyl C | di-O-alkyl C | Aromatic C | Phenolic C |  |
| Macroaggregate | CM | 0.50 (0.02) | 0.37 (0.05) | 1.03 (0.17) | 0.37 (0.01) | 0.71 (0.06) | 0.30 (0.04) | 0.47 (0.05) |
|  | HCM | 0.27 (0.01) | 0.18 (0.02) | 0.61 (0.07) | 0.21 (0.02) | 0.41 (0.02) | 0.14 (0.02) | 0.28 (0.03) |
|  | NPK | 0.13 (0.01) | 0.09 (0.01) | 0.29 (0.01) | 0.10 (0.01) | 0.21 (0.02) | 0.06 (0.01) | 0.12 (0.01) |
|  | NP | 0.11 (0.02) | 0.10 (0.01) | 0.34 (0.03) | 0.13 (0.01) | 0.23 (0.02) | 0.08 (0.01) | 0.12 (0.02) |
|  | CK | 0.08 (0.01) | 0.05 (0.00) | 0.16 (0.02) | 0.06 (0.01) | 0.14 (0.03) | 0.03 (0.00) | 0.06 (0.01) |
| Microaggregate | CM | 0.69 (0.04) | 0.50 (0.02) | 1.26 (0.06) | 0.44 (0.02) | 0.85 (0.04) | 0.37 (0.03) | 0.71 (0.04) |
|  | HCM | 0.65 (0.09) | 0.43 (0.09) | 1.31 (0.16) | 0.42 (0.09) | 0.96 (0.25) | 0.30 (0.12) | 0.70 (0.21) |
|  | NPK | 0.56 (0.07) | 0.32 (0.04) | 0.95 (0.07) | 0.30 (0.02) | 0.64 (0.05) | 0.24 (0.01) | 0.53 (0.03) |
|  | NP | 0.35 (0.05) | 0.32 (0.05) | 0.95 (0.08) | 0.34 (0.03) | 0.64 (0.05) | 0.25 (0.01) | 0.44 (0.05) |
|  | CK | 0.48 (0.07) | 0.29 (0.02) | 0.81 (0.04) | 0.26 (0.02) | 0.68 (0.10) | 0.19 (0.03) | 0.32 (0.01) |
| Silt + clay fraction | CM | 0.25 (0.04) | 0.16 (0.02) | 0.38 (0.06) | 0.12 (0.02) | 0.24 (0.04) | 0.09 (0.02) | 0.23 (0.02) |
|  | HCM | 0.23 (0.06) | 0.14 (0.05) | 0.40 (0.13) | 0.13 (0.04) | 0.24 (0.06) | 0.07 (0.02) | 0.23 (0.08) |
|  | NPK | 0.22 (0.01) | 0.13 (0.02) | 0.33 (0.01) | 0.10 (0.01) | 0.20 (0.03) | 0.06 (0.00) | 0.18 (0.02) |
|  | NP | 0.12 (0.02) | 0.09 (0.01) | 0.24 (0.06) | 0.08 (0.01) | 0.15 (0.03) | 0.05 (0.01) | 0.12 (0.01) |
|  | CK | 0.15 (0.02) | 0.07 (0.01) | 0.17 (0.01) | 0.04 (0.00) | 0.09 (0.01) | 0.02 (0.00) | 0.09 (0.01) |

Numbers in parentheses denote the standard error of the means (*n* = 4).

**Table S4**. Particle size distribution (%) in bulk soils and aggregates as affected by long-term application of compost and fertilizers.

| Aggregate | Treatment | Coarse sand | Fine sand | Silt | Clay |
| --- | --- | --- | --- | --- | --- |
| Bulk soil | CM | 0.2 | 47.4 | 34.5 | 15.6 |
|  | HCM | 0.3 | 54.2 | 29.9 | 15.6 |
|  | NPK | 0.3 | 53.1 | 31.9 | 14.7 |
|  | NP | 0.2 | 50.3 | 31.9 | 15.1 |
|  | CK | 0.1 | 50.5 | 35.3 | 14.0 |
| Macroaggregate | CM | 0.7 | 55.1 | 33.0 | 11.2 |
|  | HCM | 0.9 | 56.1 | 28.5 | 14.5 |
|  | NPK | 0.8 | 59.3 | 28.0 | 11.9 |
|  | NP | 1.3 | 56.3 | 30.3 | 12.0 |
|  | CK | 1.5 | 51.1 | 28.2 | 19.1 |
| Microaggregate | CM | 0.0 | 59.3 | 30.1 | 10.6 |
|  | HCM | 0.1 | 55.1 | 32.2 | 12.6 |
|  | NPK | 0.0 | 62.7 | 33.9 | 3.4 |
|  | NP | 0.0 | 53.1 | 33.1 | 13.8 |
|  | CK | 0.0 | 58.6 | 28.2 | 13.2 |
| Silt + clay fraction | CM | 0.0 | 8.8 | 45.0 | 46.3 |
|  | HCM | 0.0 | 1.8 | 51.8 | 46.4 |
|  | NPK | 0.0 | 1.0 | 52.6 | 46.4 |
|  | NP | 0.0 | 7.8 | 45.4 | 46.8 |
|  | CK | 0.0 | 4.2 | 61.4 | 34.4 |

**Table S5.** Ratios of clay/OC, silt_20μm_/OC and (clay+silt_20μm_)/OC in aggregates as affected by long-term application of compost and fertilizers.

| Treatment | Macroaggregate | | | Microaggregate | | | Silt + clay fraction | | |
| --- | --- | --- | --- | --- | --- | --- | --- | --- | --- |
|  | Clay/OC | Silt_20μm_/OC | (Clay+silt_20μm_)/OC | Clay/OC | Silt_20μm_/OC | (Clay+silt_20μm_)/OC | Clay/OC | Silt_20μm_/OC | (Clay+silt_20μm_)/OC |
| CM | 9.2 | 10.7 | 19.9 | 12.1 | 16.1 | 28.1 | 44.0 | 32.4 | 76.4 |
| HCM | 12.3 | 13.5 | 25.8 | 16.7 | 16.4 | 33.1 | 63.4 | 59.1 | 122.5 |
| NPK | 10.2 | 11.0 | 21.2 | 6.8 | 30.2 | 36.9 | 84.0 | 69.4 | 153.4 |
| NP | 10.9 | 15.2 | 26.1 | 28.6 | 33.6 | 62.2 | 116.4 | 97.0 | 213.4 |
| CK | 28.9 | 25.4 | 54.3 | 31.2 | 24.3 | 55.5 | 109.1 | 157.1 | 266.2 |
| Average | 14.3 (7.4) | 15.2 (5.4) | 29.5 (12.6) | 19.1 (9.4) | 24.1 (7.1) | 43.2 (13.3) | 83.4 (27.2) | 83.0 (42.4) | 166.4 (66.9) |

Numbers in parentheses denote the standard error of the means (*n* = 5).

**Table S6.** Path analysis of the content of methoxyl C in microaggregate, phenolic C in microaggregate, alkyl C in silt + clay fraction and methoxyl C in silt + clay fraction on macroaggregation.

| Model | Variable | Macroaggregation | | | |
| --- | --- | --- | --- | --- | --- |
|  |  | *R* Square (*P*) | Unstandardized coefficients | Standardized coefficients | *P* value |
| 1 | Constant | 0.986 (0.001) | 3.535 |  | 0.000 |
|  | Methoxyl C in microaggregate |  | 1.602 | 0.993 | 0.001 |
| 2 | Constant | 0.987 (0.013) | 3.472 |  | 0.003 |
|  | Methoxyl C in microaggregate |  | 1.800 | 1.115 | 0.085 |
|  | Phenolic C in microaggregate |  | -0.194 | -0.126 | 0.753 |
| 3 | Constant | 0.990 (0.129) | 3.532 |  | 0.048 |
|  | Methoxyl C in microaggregate |  | 2.068 | 1.282 | 0.253 |
|  | Phenolic C in microaggregate |  | -0.299 | -0.194 | 0.743 |
|  | Alkyl C in silt + clay fraction |  | -0.144 | -0.114 | 0.691 |
| 4 | Constant | 1.000 (-) | 3.819 |  |  |
|  | Methoxyl C in microaggregate |  | 0.183 | 0.113 | - |
|  | Phenolic C in microaggregate |  | 2.180 | 1.416 | - |
|  | Alkyl C in silt + clay fraction |  | 1.207 | 0.955 | - |
|  | Methoxyl C in silt + clay fraction |  | -1.679 | -1.442 | - |

Numbers in parentheses denote the *P* value for *R*^2^.
